# Studies on the Antidiabetic Activities of *Cordyceps militaris* Extract in Diet-Streptozotocin-Induced Diabetic Sprague-Dawley Rats

**DOI:** 10.1155/2014/160980

**Published:** 2014-03-11

**Authors:** Yuan Dong, Tianjiao Jing, Qingfan Meng, Chungang Liu, Shuang Hu, Yihang Ma, Yan Liu, Jiahui Lu, Yingkun Cheng, Di Wang, Lirong Teng

**Affiliations:** ^1^College of Life Science, Jilin University, Changchun 130012, China; ^2^College of Clinical Medicine, Jilin University, Changchun 130012, China; ^3^College of Life Science, Zhuhai College of Jilin University, Zhuhai 519000, China

## Abstract

Due to substantial morbidity and high complications, diabetes mellitus is considered as the third “killer” in the world. A search for alternative antidiabetic drugs from herbs or fungi is highly demanded. Our present study aims to investigate the antidiabetic activities of *Cordyceps militaris* on diet-streptozotocin-induced type 2 diabetes mellitus in rats. Diabetic rats were orally administered with water extract or alcohol extract at 0.05 g/kg and 2 g/kg for 3 weeks, and then, the factors levels related to blood glucose, lipid, free radicals, and even nephropathy were determined. Pathological alterations on liver and kidney were examined. Data showed that, similar to metformin, *Cordyceps militaris* extracts displayed a significant reduction in blood glucose levels by promoting glucose metabolism and strongly suppressed total cholesterol and triglycerides concentration in serum. *Cordyceps militaris* extracts exhibit antioxidative effects indicated by normalized superoxide dismutase and glutathione peroxidase levels. The inhibitory effects on blood urea nitrogen, creatinine, uric acid, and protein revealed the protection of *Cordyceps militaris* extracts against diabetic nephropathy, which was confirmed by pathological morphology reversion. Collectively, *Cordyceps militaris* extract, a safe pharmaceutical agent, presents excellent antidiabetic and antinephropathic activities and thus has great potential as a new source for diabetes treatment.

## 1. Introduction

Diabetes mellitus (DM) is characterized by chronic hyperglycaemia which is resulted by the defects of insulin secretion or action. Diabetes patients suffer with a series of metabolic disorders in carbohydrate, fat, and proteins [1]. Noninsulin-dependent diabetes mellitus (NIDDM), caused by insulin resistance, is known as the most common form of diabetes (type 2 diabetes) [2]. According to statistics, till 2025, 8 billion people in the whole world will suffer with type 2 diabetes. Additionally, various complications including cardiovascular disease, nephropathy, neuropathy, retinopathy, and hyperlipemia are observed in most diabetes patients [3].

As a cosmopolitism tough problem, no satisfactory therapeutic regimen can cure diabetes although most of them normalize blood glucose and fat levels, possess hypotensive activity, and improve microcirculation [4]. Traditional therapy only focuses on pancreatic islet function recovery and blood glucose regulation, which fails to control the diabetic complications [5]. As reported previously, insulin injection and some oral antihyperglycemic agents, such as metformin and pioglitazone, display undesirable adverse effects [6]. Pioglitazone induces hepatocellular-cholestatic liver injury and metformin causes diarrhea and nausea or vomiting [7]. Additionally, weight gain, hypoglycemia, edema, gastrointestinal disturbances, and insulin resistance are observed in diabetes patients who receive long-term insulin treatment [8]. Meanwhile, diabetes mellitus requires lifelong medication, and the economic burden of patients should receive attention [9]. Due to the limitation of existing antidiabetic agents, a search for alternative treatment is highly demanded.

Herbal medicine turns out to be a valuable reservoir for novel drugs due to its few side effects [10]. Amount of research demonstrated that natural products possess antidiabetic activity with less adverse effects and show great auxiliary therapeutic effect on complications [11, 12].* Cordyceps militaris*, an anamorph of* Cordyceps sinensis*, is advertised as a Chinese herb with antioxidant [13], immunomodulatory [14], anticancer, and anti-inflammatory pharmacological [15] effects.* Cordyceps* polysaccharides, the richest and most important activity component, display a hypoglycemic activity [16]. Additionally, several studies have shown that water extracts of* Cordyceps militaris* possess notable activity via increment of insulin secretion and cholinergic activation in normal Wistar rats [17, 18]. Excitingly, separated research finds that* Cordyceps militaris* can be used for kidney protection [19]. However, the regulatory effects of polysaccharide-enriched fraction of* Cordyceps militaris* on Sprague-Dawley rats with diabetes have not been reported yet.

We therefore hypothesized that* Cordyceps militaris* extracts may show antidiabetic, hypolipidemic, and even antinephritic effects. To test this hypothesis, the present study aims to investigate the related biological activities of* Cordyceps militaris* extracts via* in vivo* experiments. After treatment with polysaccharide-enriched fractions of* Cordyceps militaris,* the changes of serum fasting glucose levels, pyruvate kinase activity, triglyceride (TG), and total cholesterol in experimental diabetic Sprague-Dawley rats were detected. Several indexes associated with oxidation resistance and hypolipidemic activity were also determined. Furthermore, the therapeutic effects of* Cordyceps militaris* extracts on diabetic nephropathy were detected through histopathologic morphology observation and four indexes analysis including blood urea nitrogen (BUN), uric acid (UA), creatinine, and urine protein.

## 2. Methods

### 2.1. Submerged Fermentation of* Cordyceps militaris*

*Cordyceps militaris* (NBRC9787; obtained from National Biological Resource Center, Japan) was cultured in a rotary shaker incubator (10 L, Biostat B; Germany) at 150 rpm for 5 days and the cultured temperature was 26°C. The cultured medium was as follows: glucose, 20 g/L; peptone, 10 g/L; yeast extract powder, 18 g/L; KH_2_PO_4_, 3 g/L; MgSO_4_·7H_2_O, 3 g/L; (NH_4_)_2_SO_4_, 10 g/L; ZnCl_2_, 0.01 g/L; Vitamin B_1_, 0.24 g/L. The mycelium pellets were harvested and lyophilized for further using.

### 2.2. *Cordyceps militaris* Extract Preparation

As reported previously [20], the water and the alcohol extract from* Cordyceps militaris* were prepared as follows: 100 g mycelial powder was extracted two times in double distilled water at 80°C for 3 h. After centrifuging at 5000 rpm for 10 min, using Sevag reagent [V (n-butanol) : V (chloroform) = 1 : 4, 50 mL], the proteins that existed in the extracts were removed [21]. After concentration, the water extract (WE) was freeze-dried and stored in vacuum environment. Similarly, the alcohol extract (AE) was prepared using alcohol distillation at 60°C for 3 h followed by proteins removing and freeze-drying. The content of the total polysaccharides was 163 ± 2.32 mg/g in WE and 104 ± 1.27 mg/g in AE.

### 2.3. *In Vivo* Experiment in Animal Model of Diabetes

Experimental protocol was approved by the Lab Animal Centre of Jilin University (licence number SCXK-(JI) 2006-0001). Sprague-Dawley male rats weighing 120–140 g were housed in groups of two in clear plastic cages and maintained on a 12 h light/dark cycle (lights on 07:00–19:00 h) at 23 ± 1°C with water and food available* ad libitum*.

**Figure 1:**
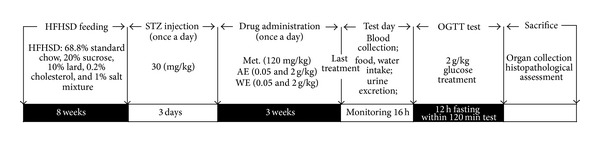
The experimental protocol for diet-STZ-induced diabetic rat model establishment and drug administration. Details are described in Section 2.

The experimental protocol for diabetic rat model establishment and drug administration was shown in Figure 1. To produce experimental model of diabetes, 42 male Sprague-Dawley rats were administrated with a modified high fat high sucrose diet (HFHSD; 68.8% standard chow, 20% sucrose, 10% lard, 0.2% cholesterol, and 1% salt mixture; purchased from the Lab Animal Centre of Jilin University, Jilin, China) [22] for 8 weeks followed by the injection of 30 mg/kg streptozotocin (STZ) for 3 days (i.p., once per day) [23]. During the experiment, 5% glucose solution was fed to rats 4 h after STZ injection to prevent hypoglycaemia. Rats with fasting serum glucose levels between 11 mmol/L and 26 mmol/L were identified as severe diabetic groups for further study [24]. Another 7 male Sprague-Dawley rats feeding with normal diet for 8 weeks and injected with citrate buffer for 3 days served as control group (CT) which were treated with normal saline orally for another 3 weeks. All diet-STZ-induced diabetic rats were separated for 6 groups randomly as follows and received drug administration for 3 weeks (once a day):

 diabetic model group (DM; *n* = 7): treatment with normal saline orally;
 metformin (DH) group (*n* = 7): treatment with 120 mg/kg metformin orally;
 low dose AE treated group (*n* = 7): treatment with 0.05 g/kg AE orally;
 high dose AE treated group (*n* = 7): treatment with 2 g/kg AE orally;
 low dose WE treated group (*n* = 7): treatment with 0.05 g/kg WE orally;
 high dose WE treated group (*n* = 7): treatment with 2 g/kg WE orally.

After 3-week treatment, food intake, water intake, and urine excretion in all rats were monitored within 16 h. Blood and urine samples were collected, and the fasting serum glucose, pyruvate kinase (PK), superoxide dismutase (SOD), glutathione peroxidase (GSH-Px), triglycerides (TG), total cholesterol, blood urea nitrogen (BUN), uric acid (UA), creatinine, and urine protein levels were determined. All the assay kits were obtained from Nanjing Biotechnology Co. Ltd. (Nanjing, China). After oral glucose tolerance test, animals were sacrificed by administration of 200 mg/kg pentobarbital; meanwhile, liver and kidney were collected and fixed in 4% paraformaldehyde.

### 2.4. Oral Glucose Tolerance Test (OGTT)

As shown in Figure 1, after 3-week treatment in diet-STZ-induced diabetic rats, an oral glucose tolerance test (OGTT) was performed. After a 12 h fast, all the experimental rats were received physiological saline, metformin, AE, or WE, respectively, as described above; 30 min later, 2 g/kg of glucose was orally given to all the rats. Blood samples were collected at 0, 30, 60, and 120 min to detect the blood glucose levels using Glucose Assay Kit (Nanjing Biotechnology Co. Ltd., Jiangsu, China). Calculation of the area under the blood glucose curve (AUC) was made according to ((I)) [25]:

(I)AUC=(basal  glycaemia+glycaemia  0.5 h)×0.25+(glycaemia  0.5 h+glycaemia  1 h)×0.25+(glycaemia  1 h+glycaemia  2 h)×0.5.

### 2.5. Histopathological Examination

Collected tissues were immerged in 4% paraformaldehyde for 48 h and then dehydrated in gradient ethanol (50%, 70%, 80%, 90%, 95%, and 100%) step by step. Samples were immerged in xylene for 30 min and incubated with first paraffin at 65°C overnight. After embedding in wax, tissues were cut into serial sections at 5 *μ*m thickness using microtome (Leica, Germany) and spread over microscopy slides. Sections were deparaffinized with fresh xylene for 10 min, hydrated with gradient ethanol (100%, 90%, 80%, and 70%), and then washed with double distilled water for three times. The sections were analyzed via haematoxylin and eosin staining (H&E staining) [26] and examined by a light microscope digital camera (Nikon Instruments, Tokyo, Japan).

### 2.6. Statistical Analysis

All values were expressed as mean ± SD. One-way analysis of variance (ANOVA) was used to detect statistical significance followed by post hoc multiple comparisons (Dunn's test). A value of *P* < 0.05 was considered to be significant.

## 3. Results

### 3.1. Bodyweight, Food and Water Intakes, and the Urine Excretion Monitoring

**Figure 2:**
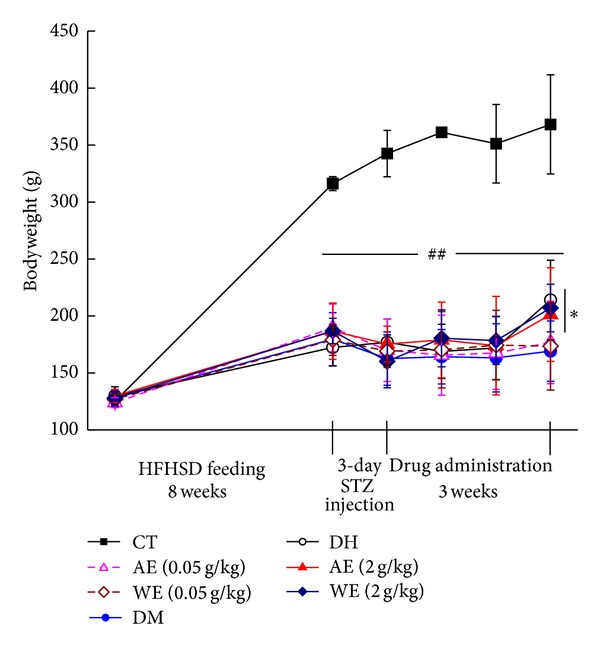
Bodyweight changes in all separated experimental groups during the whole process. Data are expressed as mean ± SD (*n* = 7) and analyzed using one-way ANOVA followed by Dunn's test. ^##^ *P* < 0.01 versus controls. *P < 0.05 versus DM group. CT: control rats; DM: diabetic rats; DH: metformin; AE: alcohol extract; WE: water extract.

Compared with CT group, DM rats consumed more food and water (*P* < 0.05; Table 1); meanwhile, more urine excretion in DM rats was noted (*P* < 0.05; Table 1). Similar to DH-administrated rats, both AE and WE treatment at 0.05 g/kg and 2 g/kg strikingly decreased urine excretion and water intakes in diet-STZ-induced diabetic rats compared with DM rats; however, no significant changes in food intakes were observed (Table 1; *P* > 0.05). Compared with CT, the growth of diet-STZ-induced diabetic rats was inhibited strongly (*P* < 0.01; Figure 2); however, after 3-week 2 g/kg AE and WE and 120 mg/kg DH treatment, the bodyweight was increased significantly compared with DM group (*P* < 0.05; Figure 2, Table 1).

### 3.2. Hypoglycemic Effects of* Cordyceps militaris* Extracts in Diet-STZ-Induced Diabetic Rats

**Figure 3:**
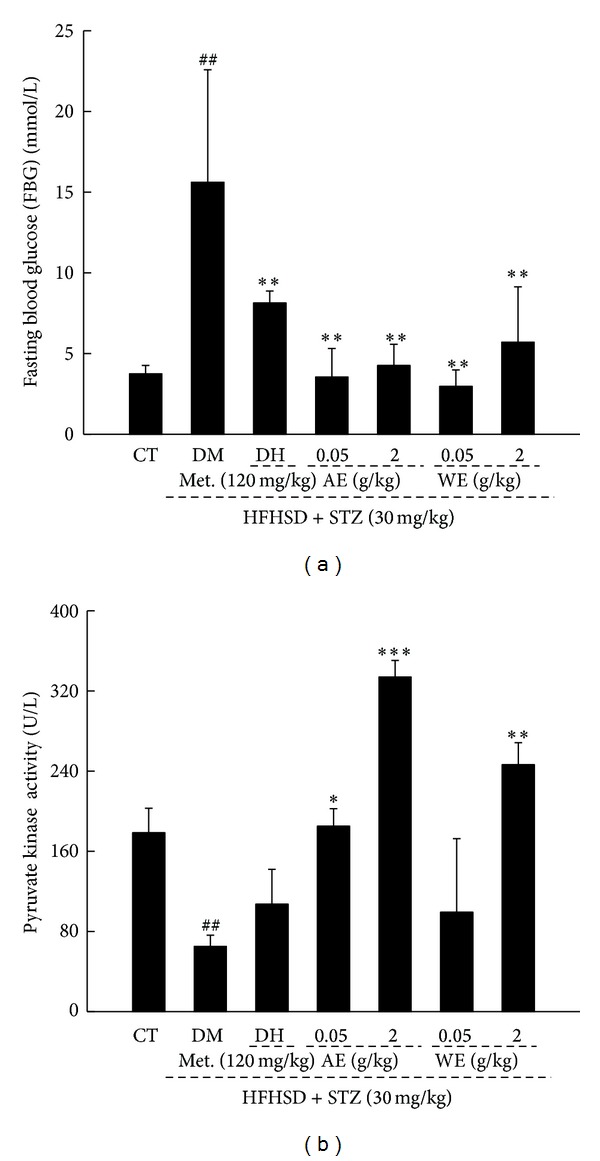
Diabetic rat model was established by 8-week HFHSD administration followed by thrice intraperitoneal injection of low doses of 30 mg/kg STZ. Nontreated rats served as control group. Diet-STZ-induced diabetic rats were treated with or without 120 mg/kg metformin (DH) and* Cordyceps militaris* extracts at various doses for another 3 weeks. The changes fasting plasma glucose level (a) and pyruvate kinase activity (b) were determined. Data are expressed as mean ± SD (*n* = 7) and analyzed using one-way ANOVA followed by Dunn's test. ^##^ *P* < 0.01 versus controls, *P < 0.05, **P < 0.01, and ***P < 0.001 versus DM group. CT: control rats; DM: diabetic rats; AE: alcohol extract; WE: water extract.

To evaluate the hypoglycemic effects of* Cordyceps militaris* extracts, the changes in fasting blood glucose (FBG) levels and PK activity were measured. Fasting blood glucose concentration in DM rats was 11.1 mmol/L higher than that of CT; while, 120 mg/kg DH and 0.05 g/kg AE and WE treatment resulted in a 46.1%, 86.3%, and 85.2% reduction, respectively (*P* < 0.01; Figure 3(a)). AE and WE administration markedly enhanced the lower PK activity in diet-STZ-induced diabetic rats (*P* < 0.05; Figure 3(b)). Especially, 2 g/kg AE treatment increased nearly 4-fold PK activity compared with DM rats (*P* < 0.001; Figure 3(b)).

### 3.3. Oral Glucose Tolerance Test

**Figure 4:**
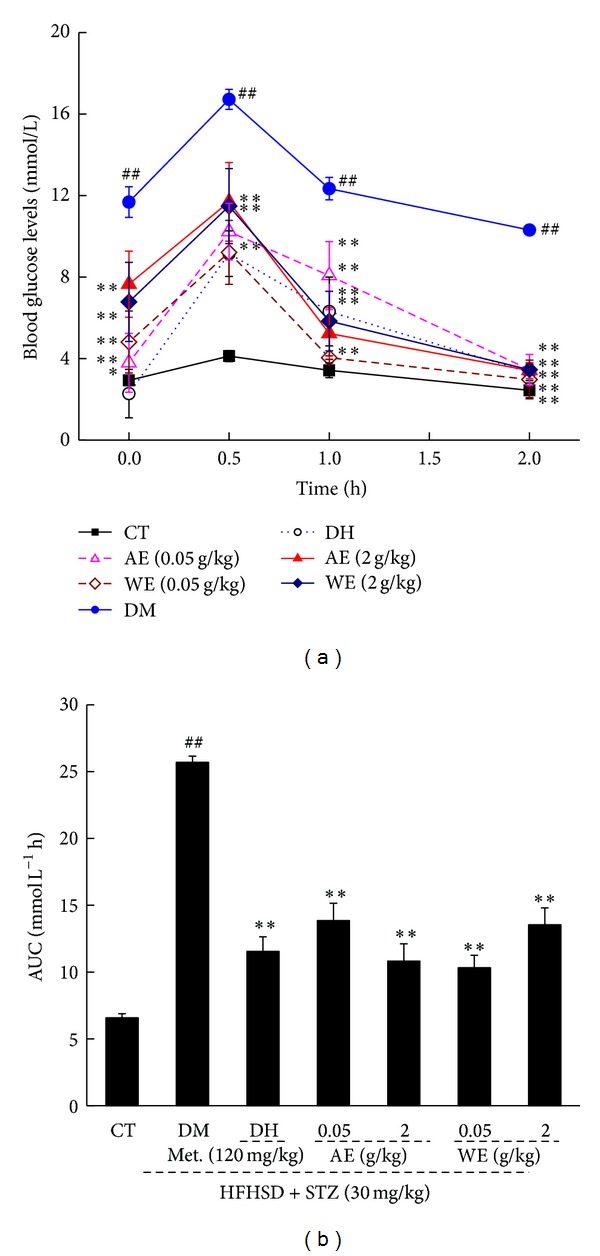
Blood glucose level (a) and AUC of OGTT (2 g glucose kg^−1^ BW) (b) in normal and diabetic rats in OGTT experiment. Data are expressed as mean ± SD (*n* = 7) and analyzed using one-way ANOVA followed by Dunn's test. ^##^ *P* < 0.01 versus controls, **P < 0.01 versus DM group. CT: control rats; DM: diabetic rats; AE: alcohol extract; WE: water extract.

In order to avoid false positive obtained from FBG, OGTT was performed as second diagnostic indices [27]. Compared with CT rats, dramatically higher fasting blood glucose concentration was noted in diet-STZ-induced diabetic rats from 0 min up to 120 min (*P* < 0.05; Figure 4(a)) indicating an impaired glucose tolerance (IGT) state. AE and WE significantly prevented the blood glucose levels shooting up, especially at 60 min and 120 min (*P* < 0.05; Figure 4(a)).

The calculated AUC values for glucose response during the OGTT revealed a striking increment in DM group (25.69 ± 0.46 mmol L^−1 ^h) compared with CT group (6.58 ± 0.31 mmol L^−1 ^h) (*P* < 0.01; Figure 4(b)). However, compared with DM group, AE and WE treatment at 0.05 g/kg and 2 g/kg showed a significant reduction in AUC (*P* < 0.01; Figure 4(b)).

### 3.4. Hypolipidemic Effects of* Cordyceps militaris* Extracts in Diet-STZ-Induced Diabetic Rats

**Figure 5:**
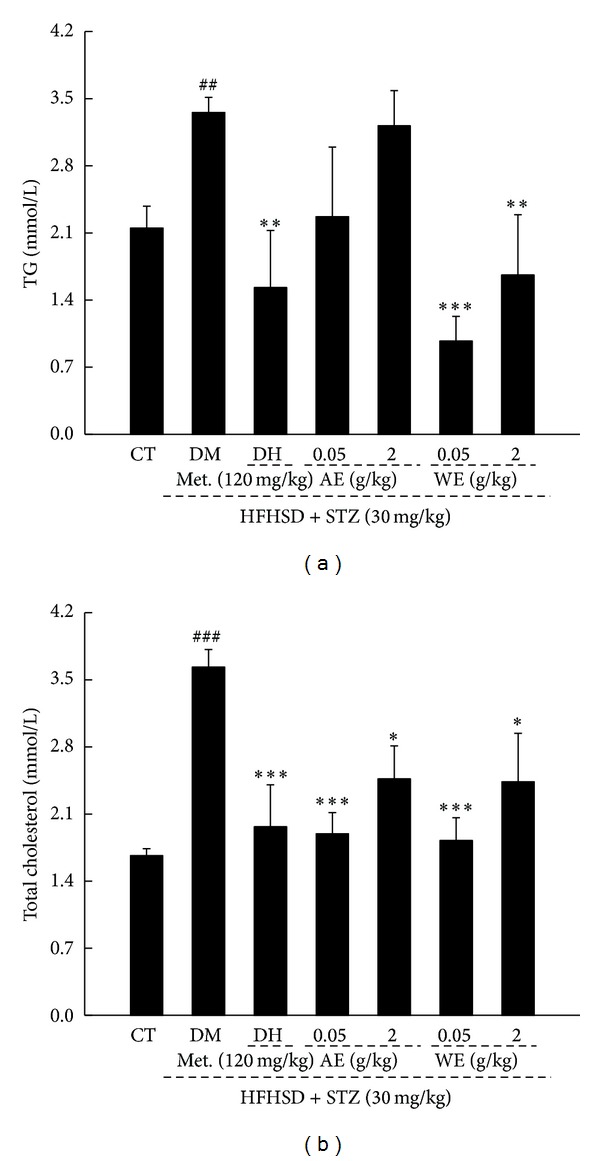
Both AE and WE treatment significantly reduce the levels of serum TG (a) and total cholesterol (b) in diet-STZ-induced diabetic rats. Data are expressed as mean ± SD (*n* = 7) and analyzed using one-way ANOVA. ^##^ *P* < 0.01 and ^###^ *P* < 0.001 versus controls, **P < 0.01 and ***P < 0.001 versus DM group. CT: control rats; DM: diabetic rats; AE: alcohol extract; WE: water extract; TG: triglycerides.

Hyperlipidemia is a common accompanied disease related to diabetes [28]. Data showed that 0.05 g/kg WE strikingly reduced serum TG concentration to 72.43% and total cholesterol concentration to 55.02% compared with DM rats (*P* < 0.01; Figure 5). AE treatment suppressed high total cholesterol concentration up to 53.42%; however, no inhibitory effect on TG concentration was noted (*P* < 0.01; Figure 5).

### 3.5. Antioxidative Effects of* Cordyceps militaris* Extracts in Diet-STZ-Induced Diabetic Rats

**Figure 6:**
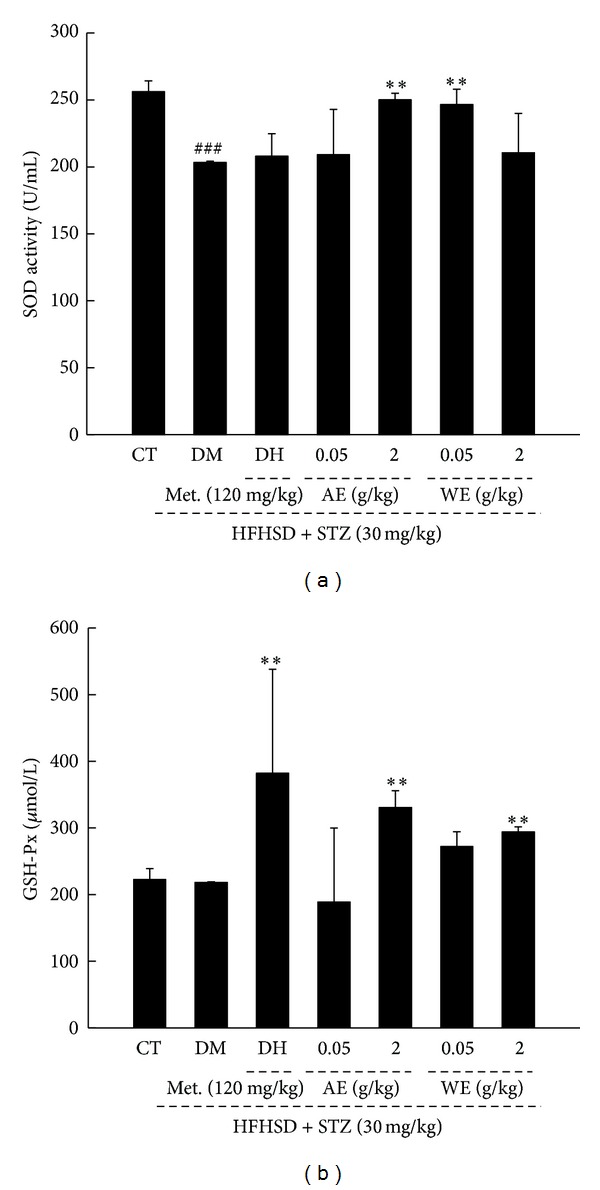
Diet-STZ-induced diabetic rats were treated with or without 120 mg/kg metformin (DH) and* Cordyceps militaris* extracts at various doses for 3 weeks. The serum SOD activity (a) and serum GSH-Px level (b) were detected. Data are expressed as mean ± SD (*n* = 7) and analyzed using one-way ANOVA followed by Dunn's test. ^###^ *P* < 0.001 versus controls, *P < 0.05 and **P < 0.01 versus DM group. CT: control rats; DM: diabetic rats; AE: alcohol extract; WE: water extract; SOD: superoxide dismutase; GSH-Px: glutathione peroxidase.

Oxidative stress is considered as a major pathogenesis in diabetes-related complications [29]. Serum SOD and GSH-Px levels were detected; among them, only abnormal SOD activity was noted in DM rats (*P* < 0.05; Figure 6(a)). AE and WE treatment resulted in an increment of SOD activity and GSH-Px level compared with DM rats (*P* < 0.05; Figure 6(b)). Meanwhile, DH only increased serum GSH-Px level in diabetic rats (*P* < 0.05; Figure 6(b)).

### 3.6. Antidiabetic Nephropathic Effects of* Cordyceps militaris* Extracts in Diet-STZ-Induced Diabetic Rats

**Figure 7:**
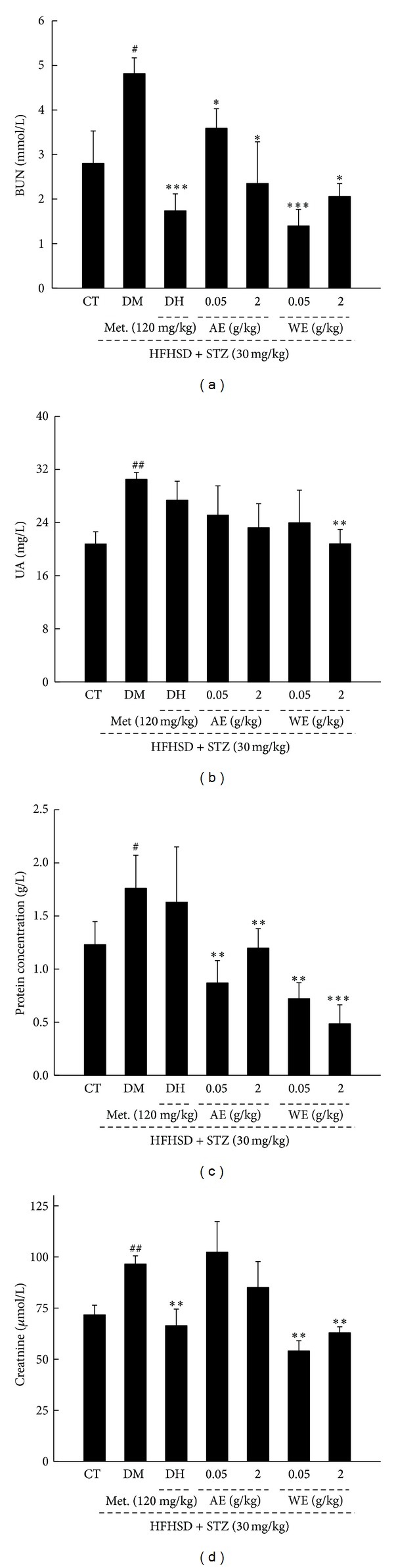
Diet-STZ-induced diabetic rats were treated with or without 120 mg/kg metformin (DH) and* Cordyceps militaris* extracts at various doses for 3 weeks. Serum BUN (a), UA (b), protein concentration (c), and creatinine (d) levels in all groups were detected. Data are expressed as mean ± SD (*n* = 7) and analyzed using one-way ANOVA followed by Dunn's test. ^##^ *P* < 0.01 versus controls, **P < 0.01 versus DM group. CT: control rats; DM: diabetic rats; DH: metformin; AE: alcohol extract; WE: water extract; BUN: blood urea nitrogen; UA: uric acid.

The levels of serum BUN, UA, creatinine, and urine protein, considered as sensitive indexes for kidney injury [30], were enhanced significantly in diet-STZ-induced diabetic rats (*P* < 0.05; Figure 7). Both AE and WE treatment reduced nearly twofold of serum BUN and protein concentration compared with DM rats (*P* < 0.05; Figures 7(a) and 7(c)). Additionally, only WE treatment suppressed the high UA and creatinine levels in serum (*P* < 0.05; Figures 7(b) and 7(d)). Interestingly, the antinephropathic effect of* Cordyceps militaris* extracts is much better than DH which only suppressed serum BUN and creatinine levels in diet-STZ-induced diabetic rats (*P* < 0.05; Figures 7(a) and 7(d)).

### 3.7. Histopathological Analysis

**Figure 8:**
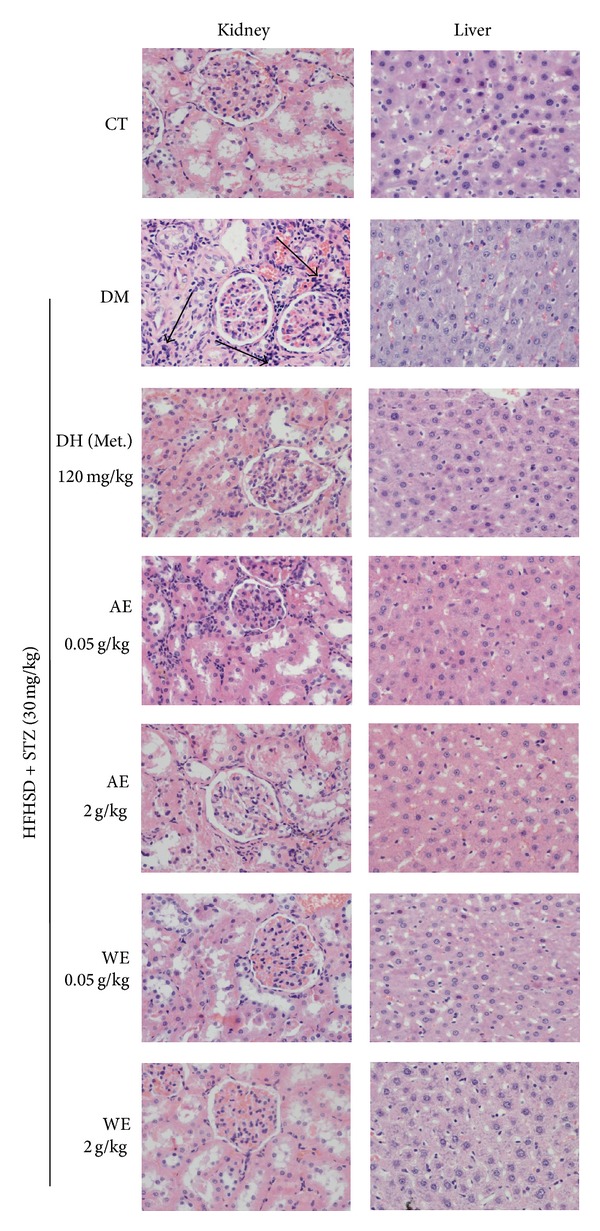
Histopathological analysis in liver and kidney collected from all experimental rats through H&E staining (x400). Arrows show regions of inflammatory cell infiltration in renal interstitium. CT: control rats; DM: diabetic rats; DH: metformin; AE: alcohol extract; WE: water extract.

Histopathological sections were performed to examine the* in vivo* toxicity of* Cordyceps militaris* extracts and further confirm their antinephropathic effects. No significant differences among experimental groups were observed in liver tissues indicating* Cordyceps militaris* is safe for animal treating (Figure 8). Moreover, data revealed that STZ caused severe injury in kidney. A large amount of inflammatory cell infiltration in renal interstitium and the atrophy of renal tubule cells was noted in DM group, whereas pathological morphology was reversed after 3-week DH, AE, and WE treatment (Figure 8).

## 4. Discussion

Diabetes has become the third “killer” in the world following cancer, cardiovascular and cerebrovascular diseases. Due to various pathologic changes, amount of diabetic complications occur in blood vessels, cranial and peripheral nerves, and skin [31]. Retinopathy and nephropathy are also considered as high frequency complications caused by the abnormal thickening of the basement membrane in capillaries [32]. The antihyperglycemic and antidyslipidemic effects of* Cordyceps militaris* water extracts have already been studied in db/db mice [33]. As reported previously, the polished rice cultivated with* Cordyceps militaris* reduces blood glucose levels and shows antioxidant effects in STZ-induced diabetic rats [27]. In our present study, the hypoglycemic effects related to fasting blood glucose levels of* Cordyceps militaris* extracts were confirmed. Furthermore, the hypolipidemic, antioxidative, and antidiabetic nephropathic effects of* Cordyceps militaris* extracts were observed in diet-STZ-induced diabetic rats. Through histopathological analysis in liver tissue,* Cordyceps militaris* was confirmed as a pharmacological safe agent.

In our present study, the results showed that both WE and AE possess hypoglycemic effect similar to that of DH, and this effect may be partially related to the increment of glucose absorption indicated by the enhancement of PK activity [34]. PK is the rate-limiting enzyme of glycolytic pathway which can promote the metabolism of sugar [35]. Due to the disorder of lipid metabolism [36], serum concentrations of total cholesterol and TG were enhanced significantly in DM rats; in contrast, both WE and AE normalized plasma lipid and lipoprotein profile.* Cordyceps militaris* extracts may prevent the accumulation of fatty acid in liver [37]. Since* Cordyceps militaris* extracts displayed no stimulating effect on insulin (data not shown), their antidiabetic effect may not be related to insulin secretion. Previous study reports that* Cordyceps militaris* water extract decreases fasting serum glucose levels by increasing glucose disposal rates and fraction velocity of glycogen synthase in 90% pancreatectomized rats [38]. Further study will be performed to confirm this result.

Increased glucose levels result in glucose autooxidation and autooxidative glycosylation of proteins [27]. Due to the unbalanced producing and scavenging, free radicals were accumulated in diabetic patients, which played a role in the pathogenesis of the long-term complications of human diabetes [39]. A deficiency of the antioxidant activity of SOD and GSH-Px in diabetes mellitus is related to higher concentration of peroxide [40]. Our data demonstrated that both AE and WE normalized SOD activity and enhanced GSH-Px levels compared with DM group. Our finding is consistent with other researches reporting that* Cordyceps militaris* extracts reduce reactive oxygen species (ROS) and nitrogen species (RNS) generation caused by high glucose [41].

Furthermore, diabetic nephropathy is reported as a serious microvascular complication of diabetes [42]. BUN, UA, urine protein, and creatinine are traditional indexes for kidney damage [43].* Cordyceps militaris* extracts displayed a strong inhibitory effect on BUN, UA, creatinine, and urine protein levels compared with DM rats. Interestingly, mesangial expansion, mesangial hypercellularity, and a thickened glomerular basement membrane, known as the morphological indicators of nephropathy [44], were not obvious in model group. However, a large amount of inflammatory cell infiltration in renal interstitium and the atrophy of renal tubule cells was noted in DM rats, which were strongly reversed after DH, AE, and WE treatment. The pathological lesions of renal tubule may also be related to STZ which is responsible for renal injury [45], oxidative stress inflammation, and endothelial dysfunction [46]. Considering the model was only established for nearly two months, diabetic nephropathy related pathological symptoms might be in early phase. Although the protection of AE and WE against diabetic nephropathy has been well confirmed, the potential mechanism needs to be further investigated.

Additionally, most researches are related to the activities of* Cordyceps militaris* water extracts which promote glucose metabolism by increasing the activities of hepatic glucokinase, hexokinase, and glucose-6-phosphate dehydrogenase [47]. Being consistent with previous findings [48], AE shows poorer hypoglycemic activity compared with WE. It may be due to the better solubility and absorption of WE; however, more experiments need to be performed to investigate the difference between them. In our separated experiment, WE was purified and characterized. One main fraction entitled CMN1 with a 37842 Da molecular weight was obtained. The backbone of CMN1 is composed of (1→2)-linkages and (1→3)-linkages with a branched (1→6)-linkage and (1→4)-linkage, and its monosaccharide composition was Rha, Ara, Man, and Gal. CMN1 shows similar molecular weight and monosaccharide composition with CSP-1 isolated by Li's group [49].

Another point we would like to emphasize here is the diabetic rat model established in the experiment. As reported previously, HFHSD raising was used to develop insulin resistance model [50, 51]; meanwhile, STZ enters the pancreatic *β* cell via a glucose transporter GLUT2 and induces a mild impairment of insulin secretion which is similar to the feature of the later stage of type 2 diabetes [23]. The model is considered as a stable animal model of type 2 diabetes and usually used for pharmaceutical screening [52]. However, our results demonstrated that this diet-STZ-induced model fails to simulate all the symptoms in type 2 diabetes. Only several related complications such as nephritis were reflected in it. Furthermore, no insulin increment was observed in DM group (data not shown). Although the increment of TG level is considered as a result of insulin resistance, it may also be related to blood glucose enhancement and fatty acid metabolism [37]. More experiments will be performed to study the unexpected data obtained in diet-STZ-induced diabetic rat model.

Altogether, by using diet-STZ-induced diabetic rats, the antidiabetic activity of* Cordyceps militaris* extracts has been confirmed in our present study.* Cordyceps militaris* extracts also show antinephropathic and antioxidative properties indicating the therapeutic effects on diabetic complications.* Cordyceps militaris* extract, a safe pharmaceutical agent, has great potential as a new source for diabetes treatment.

## Figures and Tables

**Table 1:** Results on the bodyweight gain, food intake, water intake, and urine excretion in each experimental group.

Group	Dose (g/kg/d)	Bodyweight gain/(g)	Water intake/(mL)	Food intake/(g)	Urine excretion/(mL)
CT	—	5.58 ± 0.27	55.00 ± 10.48	9.17 ± 1.60	36.67 ± 13.70
DM	—	6.61 ± 1.29	102.00 ± 5.41^b^	16.25 ± 0.50^b^	82.50 ± 5.00^b^
DH treated	0.12	37.06 ± 2.38^a^	37.50 ± 9.58^a^	4.75 ± 1.50^a^	22.50 ± 2.65^a^
AE treated	0.05	6.75 ± 0.68	54.17 ± 32.31^a^	6.33 ± 2.50	29.60 ± 3.55^a^
	2	25.82 ± 2.14^a^	50.00 ± 17.03^a^	5.41 ± 1.02	37.60 ± 19.03^a^
WE treated	0.05	38.49 ± 3.86^a^	46.67 ± 5.77^a^	7.67 ± 2.51	21.33 ± 6.11^a^
	2	13.34 ± 1.57	53.33 ± 15.27^a^	5.67 ± 0.57	56.60 ± 15.27^a^

Data are expressed as mean ± SD (*n* = 7/group) and analyzed using ANOVA followed by Dunn's test.

^a^Statistical significance compared with DM (*P* < 0.05); ^b^statistical significance compared with CT (*P* < 0.05).

CT: control rats; DM: diabetic rats; DH: metformin; AE: alcohol extract; WE: water extract.

## References

[1] Kerner W, Bruckel J (2011). Definition, diagnosis and classification of diabetes mellitus. *Diabetologie und Stoffwechsel*.

[2] Cao Z, Cooper M (2011). Pathogenesis of diabetic nephropathy. *Journal of Diabetes Investigation*.

[3] Winkler G, Hidvegi T, Vandorfi G (2011). Risk-stratified screening for type 2 diabetes in adult subjects: results from Hungary. *Diabetologia*.

[4] Levterova BA, Dimitrova DD, Levterov GE (2013). Instruments for disease-specific quality-of-life measurement in patients with type 2 diabetes mellitus—a systematic review. *Folia Medica*.

[5] Kania DS, Gonzalvo JD, Weber ZA (2011). Saxagliptin: a clinical review in the treatment of type 2 diabetes mellitus. *Clinical Therapeutics*.

[6] Fuangchan A, Sonthisombat P, Seubnukarn T (2011). Hypoglycemic effect of bitter melon compared with metformin in newly diagnosed type 2 diabetes patients. *Journal of Ethnopharmacology*.

[7] Andújar-Plata P, Pi-Sunyer X, Laferrère B (2012). Metformin effects revisited. *Diabetes Research and Clinical Practice*.

[8] Scheen A (2007). Antidiabetic agents in subjects with mild dysglycaemia: prevention or early treatment of type 2 diabetes?. *Diabetes & Metabolism*.

[9] Shyangdan D, Cummins E, Royle P (2011). Liraglutide for the treatment of type 2 diabetes. *Health Technology Assessment*.

[10] Novak M, Vetvicka V (2008). *β*-glucans, history, and the present: immunomodulatory aspects and mechanisms of action. *Journal of Immunotoxicology*.

[11] Tanaka M, Misawa E, Ito Y (2006). Identification of five phytosterols from aloe vera gel as anti-diabetic compounds. *Biological and Pharmaceutical Bulletin*.

[12] Kamtchouing P, Kahpui SM, Dzeufiet PD, Tédong L, Asongalem EA, Dimo T (2006). Anti-diabetic activity of methanol/methylene chloride stem bark extracts of *Terminalia superba* and *Canarium schweinfurthii* on streptozotocin-induced diabetic rats. *Journal of Ethnopharmacology*.

[13] Li S, Zhao K, Ji Z (2003). A polysaccharide isolated from * Cordyceps sinensis*, a traditional Chinese medicine, protects PC12 cells against hydrogen peroxide-induced injury. *Life Sciences*.

[14] Kim CS, Lee S-Y, Cho S-H (2008). *Cordyceps militaris* induces the IL-18 expression via its promoter activation for IFN-*γ* production. *Journal of Ethnopharmacology*.

[15] Paterson RR (2008). Cordyceps: a traditional traditional Chinese medicine and another fungal therapeutic biofactory?. *Phytochemistry*.

[16] Zhang G, Huang Y, Bian Y, Wong JH, Ng TB, Wang H (2006). Hypoglycemic activity of the fungi *Cordyceps militaris*, *Cordyceps sinensis*, *Tricholoma mongolicum*, and *Omphalia lapidescens* in streptozotocin-induced diabetic rats. *Applied Microbiology and Biotechnology*.

[17] Cheng YW, Chen YI, Tzeng CY (2012). Extracts of *Cordyceps militaris* lower blood glucose via the stimulation of cholinergic activation and insulin secretion in normal rats. *Phytotherapy Research*.

[18] Koh JH, Kim JM, Chang UJ, Suh H-J (2003). Hypocholesterolemic effect of hot-water extract from mycelia of * Cordyceps sinensis*. *Biological and Pharmaceutical Bulletin*.

[19] Wang Y, Yin HP, Lv XB, Wang Y, Gao H, Wang M (2010). Protection of chronic renal failure by a polysaccharide from * Cordyceps sinensis*. *Fitoterapia*.

[20] Du L, Song J, Wang H (2012). Optimization of the fermentation medium for Paecilomyces tenuipes N45 using statistical approach. *African Journal of Microbiology Research*.

[21] Yan H, Zhu D, Xu D, Wu J, Bian X (2008). A study on *Cordyceps militaris* polysaccharide purification, composition and activity analysis. *African Journal of Biotechnology*.

[22] Wang JS, Zhang W, Zhu D, Zhu X, Pang X, Qu W (2011). Hypolipidaemic and hypoglycaemic effects of total flavonoids from seed residues of *Hippophae rhamnoides* L. in mice fed a high-fat diet. *Journal of the Science of Food and Agriculture*.

[23] Frode T, Medeiros Y (2008). Animal models to test drugs with potential antidiabetic activity. *Journal of Ethnopharmacology*.

[24] Federiuk I, Casey H, Quinn M (2004). Induction of type-1 diabetes mellitus in laboratory rats by use of alloxan: route of administration, pitfalls, and insulin treatment. *Comparative Medicine*.

[25] Subramanian R, Asmawi MZ, Sadikun A (2008). *In vitroα*-glucosidase and *α*-amylase enzyme inhibitory effects of Andrographis paniculata extract and andrographolide. *Acta Biochimica Polonica*.

[26] Espada J, Valverde P, Stockert JC (1993). Selective fluorescence of eosinophilic structures in grasshopper and mammalian testis sustained with haematoxylin-eosin. *Histochemistry*.

[27] Park JH, Park NS, Lee SM, Park E (2011). Effect of dongchunghacho rice on blood glucose level, lipid profile, and antioxidant metabolism in streptozotocin-induced diabetic rats. *Food Science and Biotechnology*.

[28] Adewole S, Adenowo T, Naicker T (2011). Hypoglycaemic and hypotensive effects of ficus exasperata vahl. (moraceae) leaf aqueous extract in rats. *African Journal of Traditional, Complementary and Alternative Medicines*.

[29] Wang BS, Lee CP, Chen ZT, Yu HM, Duh P-D (2012). Comparison of the hepatoprotective activity between cultured *Cordyceps militaris* and natural * Cordyceps sinensis*. *Journal of Functional Foods*.

[30] Kumar G, Shetty A, Salimath P (2008). Modulatory effect of bitter gourd (Momordica charantia LINN.) on alterations in kidney heparan sulfate in streptozotocin-induced diabetic rats. *Journal of Ethnopharmacology*.

[31] O’Connell P, Taba M, Nomizo A (2008). Effects of periodontal therapy on glycemic control and inflammatory markers. *Journal of Periodontology*.

[32] Federation ID (2013). Diabetes and the millennium development goals International Diabetes Federation. *Diabetes Research and Clinical Practice*.

[33] Choi H, Kang M, Jeong S (2012). Effect of Dongchunghacho (*Cordyceps militaris*) on hyperglycemia and dyslipidemia in type 2 diabetic db/db mice. *Food Science and Biotechnology*.

[34] Dhawan K, Kumar S, Sharma A (2002). Suppression of alcohol-cessation-oriented hyper-anxiety by the benzoflavone moiety of Passiflora incarnata Linneaus in mice. *Journal of Ethnopharmacology*.

[35] O’Callaghan B, Koo S, Wu Y, Freake HC, Towle HC (2001). Glucose regulation of the Acetyl-CoA carboxylase promoter PI in rat hepatocytes. *Journal of Biological Chemistry*.

[36] Zhao J, Zhang W, Zhu X (2011). The aqueous extract of Asparagus officinalis L. by-product exerts hypoglycaemic activity in streptozotocin-induced diabetic rats. *Journal of the Science of Food and Agriculture*.

[37] Shih K-C, Kwok C-F, Hwu C-M (1997). Acipimox attenuates hypertriglyceridemia in dyslipidemic noninsulin dependent diabetes mellitus patients without perturbation of insulin sensitivity and glycemic control. *Diabetes Research and Clinical Practice*.

[38] Choi SB, Park CH, Choi MK, Jun DW, Park S (2004). Improvement of insulin resistance and insulin secretion by water extracts of *Cordyceps militaris*, Phellinus linteus, and Paecilomyces tenuipes in 90% pancreatectomized rats. *Bioscience, Biotechnology and Biochemistry*.

[39] Baynes JW, Thorpe SR (1996). The role of oxidative stress in diabetic complications. *Current Opinion in Endocrinology and Diabetes*.

[40] Suryawanshi NP, Bhutey AK, Nagdeote AN, Jadhav AA, Manoorkar GS (2006). Study of lipid peroxide and lipid profile in diabetes mellitus. *Indian Journal of Clinical Biochemistry*.

[41] Chu H-L, Chien J-C, Duh P-D (2011). Protective effect of *Cordyceps militaris* against high glucose-induced oxidative stress in human umbilical vein endothelial cells. *Food Chemistry*.

[42] van Dijk C, Berl T (2004). Pathogenesis of diabetic nephropathy. *Reviews in Endocrine & Metabolic Disorders*.

[43] Zhou X, Yao Y (2013). Unexpected nephrotoxicity in male ablactated rats induced by *Cordyceps militaris*: the involvement of oxidative changes. *Evidence-Based Complementary and Alternative Medicine*.

[44] Fioretto P, Mauer M (2007). Histopathology of diabetic nephropathy. *Seminars in Nephrology*.

[45] Valentovic MA, Alejandro N, Betts Carpenter A, Brown PI, Ramos K (2006). Streptozotocin (STZ) diabetes enhances benzo(*α*)pyrene induced renal injury in Sprague Dawley rats. *Toxicology Letters*.

[46] Lei Y-C, Hwang J-S, Chan C-C, Lee C-T, Cheng T-J (2005). Enhanced oxidative stress and endothelial dysfunction in streptozotocin-diabetic rats exposed to fine particles. *Environmental Research*.

[47] Kiho T, Yamane A, Hui J (1996). Polysaccharides in fungi. XXXVI.1 Hypoglycemic activity of a polysaccharide (CS-F30) from the cultural mycelium of * Cordyceps sinensis* and its effect on glucose metabolism in mouse liver. *Biological and Pharmaceutical Bulletin*.

[48] Kan W-C, Wang H-Y, Chien C-C (2012). Effects of extract from solid-state fermented * Cordyceps sinensis* on type 2 diabetes mellitus. *Evidence-Based Complementary and Alternative Medicine*.

[49] Li SP, Zhang GH, Zeng Q (2006). Hypoglycemic activity of polysaccharide, with antioxidation, isolated from cultured *Cordyceps* mycelia. *Phytomedicine*.

[50] Nakamura T, Terajima T, Ogata T (2006). Establishment and pathophysiological characterization of type 2 diabetic mouse model produced by streptozotocin and nicotinamide. *Biological and Pharmaceutical Bulletin*.

[51] Zhao S, Chu Y, Zhang C (2008). Diet-induced central obesity and insulin resistance in rabbits. *Journal of Animal Physiology and Animal Nutrition*.

[52] Zhang M, Lv XY, Li J, Xu Z-G, Chen L (2008). The characterization of high-fat diet and multiple low-dose streptozotocin induced type 2 diabetes rat model. *Experimental Diabetes Research*.

